# Characterizing Psychomotor Abilities of Male Handball Players of Different Age Categories

**DOI:** 10.5114/jhk/203954

**Published:** 2025-09-23

**Authors:** Francisco Martins, Maciej Śliż, Cíntia França, Hugo Sarmento, Élvio Rúbio Gouveia, Pedro Campos, Helder Lopes, Krzysztof Przednowek

**Affiliations:** 1 Research Unit for Sport and Physical Activity (CIDAF), Faculty of Sports Sciences and Physical Education, University of Coimbra, Coimbra, Portugal.; 2 Department of Physical Education and Sport, University of Madeira, Funchal, Portugal.; 3 LARSYS, Interactive Technologies Institute, Funchal, Portugal.; 4 Faculty of Physical Culture Sciences, Medical College, University of Rzeszów, Rzeszów, Poland.; 5 Swiss Center of Expertise in Life Course Research LIVES, Carouge, Switzerland.; 6 Department of Informatics Engineering and Interactive Media Design, University of Madeira, Funchal, Portugal.; 7 Wowsystems Informática Lda, Funchal, Portugal.; 8 Research Center in Sports Science, Health Sciences, and Human Development (CIDESD), Vila Real, Portugal.

**Keywords:** team sports, cognitive abilities, youth, Test2Drive, reaction time

## Abstract

Sports performance is highly influenced by players’ mental, emotional, and psychomotor abilities. Particularly in handball, training methods aim to maximize players’ performance by fusing physical conditioning with cognitive abilities. The aim of this research was twofold: (1) to characterize players’ psychomotor abilities according to their age categories (U14, U16, and U18), and (2) to analyze the differences between psychomotor abilities profiles according to players’ field positions. The study population comprised 75 male youth handball players (15.3 ± 1.5 years) from different competitive age groups (U14 = 20 players; U16 = 27 players; U18 = 28 players). The psychomotor abilities were evaluated using the Test2Drive system. Three tests were performed to measure psychomotor abilities: (a) a simple reaction time test (SIRT), (b) a choice reaction time test (CHORT), and (c) a spatial anticipation test (SPANT). The only statistically significant difference was observed in the percentage of correct answers in the SPANT, showing that U16 players had a higher rate of correct answers than the others. However, some trends were visible throughout the analysis: (i) the U16 group performed better in terms of reaction time in all the tests, (ii) right-wingers were always the ones who had the best percentage of correct answers, and (iii) central players had the best movement time performance. Future research should include youth players' maturation stage, body composition, and physical fitness performance to characterize their profile deeply and analyze their psychomotor abilities, considering other factors that can impact their sports performance.

## Introduction

Sports performance is generally influenced by athletes’ mental, emotional, and psychomotor skills and is crucial to achieving the highest motor manifestation indices (Hermassi et al., 2014). The extensive range of motor coordination abilities in individual players, some of them being a fast reaction, motor adjustment, time-space orientation, combining movements, and high frequency of movements, is one of the key factors that determine how practical sports training is in team sports (Gaamouri et al., 2024; Klocek et al., 2023).

In handball, the high-intensity nature of training and competition is typified by players running, changing direction with or without the ball, interacting with their opponent, and making rapid decisions when playing both offense and defense (Gómez-López et al., 2024; Krüger et al., 2014; Meletakos and Bayios, 2010; Shahbazi et al., 2011). Being an Olympic sport, handball has been significantly impacted by psycho-physical training, combining mental and physical approaches (Spieszny et al., 2024). This training method maximizes players’ sports skills and performance by fusing physical conditioning with cognitive abilities.

Sports skills are frequently defined as motor habits and the ability to use them reflexively during competition (Lech et al., 2014). Indeed, perception (receiving and identifying a stimulus), information processing (selecting the appropriate response), and action (motor reaction) comprise the three stages of the most basic motor activity scheme (Schmidt and Wrisberg, 2004). Therefore, the quality of receptor activity, the effectiveness of the central nervous system’s excitation and inhibition processes, the speed and quality of information carried out in the peripheral system, and neuromuscular coordination all affect how effective motor activity is (Klocek et al., 2023).

Psychomotor skills are the mental processes connected to humans’ physical movements, and they depend on developing specific abilities (Spieszny et al., 2024). For instance, sports like handball involve competitive settings that vary in the area, time and factors that influence the information opponents supply (Muntianu et al., 2022). Athletes with strong decision-making skills can maneuver on the field optimally. According to several authors (Abbott et al., 2005; Kida et al., 2005; Mańkowska et al., 2015), winning depends on quick reaction time due to oculomotor coordination. Reaction times were evaluated, and the results showed that winning teams had substantially better reaction times, translating into wins.

Some factors that can aid a team in winning a handball game are anticipating the opponent’s moves with or without the ball, paying attention, selecting the right maneuver, perceiving, and having high levels of sensory and motor fitness (Kida et al., 2005; Nakamoto and Mori, 2008). Players can better respond to outside inputs and modify their motions in response to events on the court because of these psychomotor skills and their high degree of eye-hand synchronization (Kioumourtzoglou et al., 1998). Players' results can also be greatly influenced by their coordination skills, which are complex abilities associated with strength, endurance, mobility, and speed (Bompa, 1995). Additionally, coordination skills are a component of physical ability for players (Baştiurea et al., 2014).

In recent years, the literature has shown that age could be related to oculomotor coordination, the ability to gather visual information about moving objects, such as opponents and the ball, response, and reaction times (Vänttinen et al., 2010). A recent systematic review concerning decision-making in youth team sports players concluded that there was a tendency for older players to make more accurate decisions in the game and to have better tactical knowledge and behavior (Silva et al., 2020). For instance, youth soccer players from Germany were divided into age groups of U12, U13, U17, and U19 (Beavan et al., 2019). Those authors revealed that the reaction time improved (i.e., shorter timespan to respond) with age. Other investigations with samples represented by youth soccer players have concluded that reaction and movement skills improve with age in youth sports categories (Hirose et al., 2002; Iida et al., 2010).

There has been some research into the psychomotor abilities of handball players (Muntianu et al., 2022; Spieszny et al., 2024; Śliż et al., 2022). However, the so-called motor tests are employed in sports practice, particularly in the early training phases. Psychomotor indices, such as simple and complex reaction time, eye-hand coordination, attention traits, visual perception, and anticipation, are more frequently assessed in professional and experienced handball players (Klocek et al., 2023; Zwierko et al., 2024). As coordinating predispositions of motor skills, these tests enable the evaluation of the indices of the nervous system's fundamental perceptual and analytical functions (Gierczuk et al., 2008). Therefore, it seems vital to understand the development of youth players in these associated psychomotor skills and better conditions for achieving sporting success. We believe there is a significant gap in the study of the influence of these abilities on youth handball players’ performance. Thus, the aim of this research was twofold: (1) to characterize players’ psychomotor abilities according to their age categories (i.e., U14, U16, and U18), and (2) to analyze the differences between psychomotor profiles according to the player’s field position.

## Methods

### Participants and Study Design

The study included 75 male adolescent handball players (15.3 ± 1.5 years). Of those, 37 participated in Portugal’s youth handball competitions and 38 in Poland’s youth handball competitions. The adolescents were divided according to their chronological age (U14 = 20 players; U16 = 27 players; U18 = 28 players), and by their field position (goalkeeper = 11; center = 13; left-back = 13; right-back = 8; left-wing = 10; right-wing = 9; pivot = 11). This cross-sectional study was conducted approximately halfway through the 2023/2024 season. The assessments took place at the clubs’ facilities 2–3 hours before the respective teams’ training sessions. Before the assessment, participants performed a familiarization session to provide them with a full understanding of the test conditions and objectives. Reaction times (RTs), movement times (MTs), and the percentage of correct answers were collected under all test conditions. All the procedures implemented in this study were conducted by an experienced research team and followed the principles outlined in the Declaration of Helsinki. The scope, project, and protocol were approved by the ethics committee of the University of Rzeszów, Rzeszów, Poland (approval code: 10/02/2020; approval date: 20 February 2020). Before the study commenced, written informed consent was obtained from all the participants and/or their legal guardians.

### Assessment of Psychomotor Abilities

**Figure 1:**
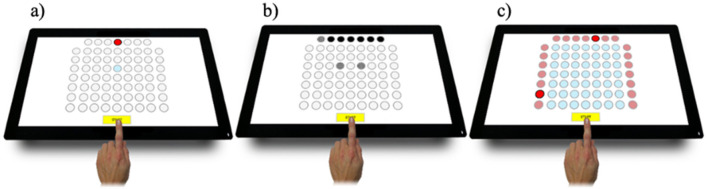
Reaction panel of the Test2Drive system: (a) SIRT, (b) CHORT, (c) SPANT.

Psychomotor abilities were evaluated using the Test2Drive system (ALTA, Siemianowice Śląskie, Poland) (Tarnowski, 2014). Three tests were performed to measure the psychomotor abilities: (a) a simple reaction time test (SIRT), (b) a choice reaction time test (CHORT), and (c) a spatial anticipation test (SPANT). The SIRT assessed reaction speed and its stability. The stimuli signaling field changed their color at precise intervals. Participants responded to the stimuli by moving their fingers from the start button to the reaction time field marked in blue. The CHORT evaluated speed and reaction adequacy. In the top signaling row, horizontal benchmarks (stimuli requiring a reaction), vertical stimuli (also requiring a reaction), and a slant benchmark (neutral stimuli that did not require a reaction) were displayed. Participants responded to the stimuli by moving their finger from the start button to one of the two reaction fields (horizontal or vertical stimulus). During the neutral stimulus, participants kept their finger on the start button. The SPANT evaluated eye-hand coordination using spatial information. Signaling fields were positioned at the test board’s top, left, and right sides. Two fields (one in a row and one in a column) turned red simultaneously. Responding to the stimulus, participants indicated the field at the intersection of the lit row and column with their fingers and then returned to the start button. The panels illustrating the (a) SIRT, (b) CHORT, and (c) SPANT tests can be seen in Figure 1 in their respective order.

### Statistical Analysis

Descriptive statistics are presented as means ± standard deviation. The normality of the data was verified using the Shapiro-Wilk test, and the results indicated the absence of normal distribution. Based on this, the Kruskal-Wallis test was conducted to examine differences in psychomotor abilities based on age groups (U14, U16, and U18) and playing positions (goalkeeper, center, left-back, right-back, left-wing, right-wing, and pivot). Effect size was calculated using Cohen criteria: 0.1 = small effect, 0.3 = medium effect, and 0.5 = large effect (Cohen, 2013). All statistical procedures were performed using the IBM SPSS Statistics software 29.0 (SPSS Inc., Chicago, IL, USA), and the significance level was set at 5%.

## Results

**Figure 2:**
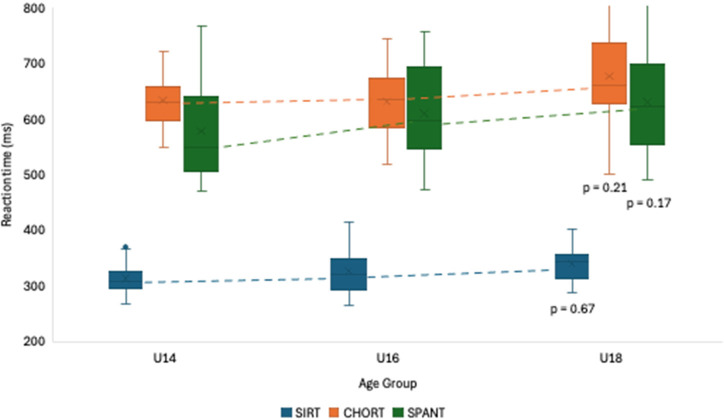
Reaction time comparisons among groups according to age.

The comparison among the age groups (i.e., U14, U16, U18) regarding reaction time abilities is presented in Figure 2. According to the results, no statistically significant differences were seen between performances. However, the results did show a visible trend that U16 players performed better in terms of reaction time in the three tests: (i) SIRT (U14 = 333.3 ± 32.4; U16 = 325.6 ± 27.9; U18 = 327.5 ± 40.4), (ii) CHORT (U14 = 678.4 ± 69.8; U16 = 638.7 ± 47.9; U18 = 640.0 ± 75.4), (iii) SPANT (U14 = 633.7 ± 95.5; U16 = 583.6 ± 80.1; U18 = 618.1 ± 101.4).

**Figure 3:**
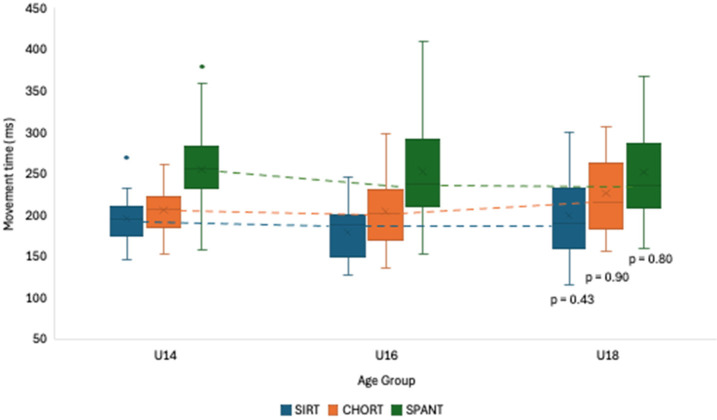
Movement time comparisons among groups according to age.

Figure 3 represents the comparison among particular age groups (i.e., U14, U16, U18) regarding movement time. The results showed no significant differences between age groups regarding movement time in the three tests. However, the U18 group showed a better performance in two out of the three tests: (i) SIRT (U14 = 194.6 ± 40.9; U16 = 194.0 ± 24.5; U18 = 186.8 ± 45.7), (ii) CHORT (U14 = 213.7 ± 38.2; U16 = 215.4 ± 57.4; U18 = 211.3 ± 46.7). Interestingly, in the SPANT it was the youngest group who performed better (U14 = 246.8 ± 55.6; U16 = 249.6 ± 50.1; U18 = 261.6 ± 66.5).

**Figure 4:**
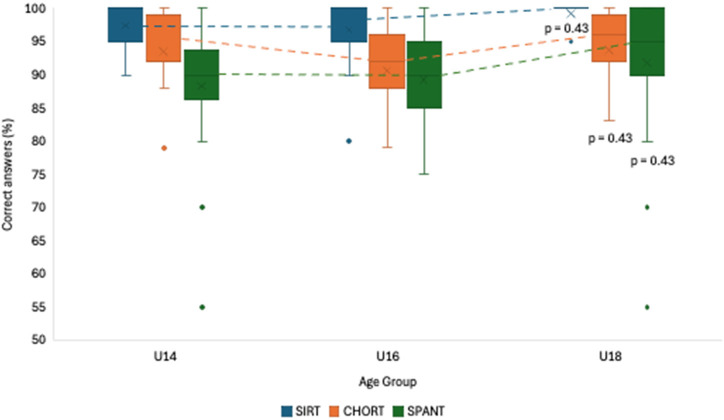
Comparison of the percentage of correct answers among groups according to age.

The comparison among the age groups (i.e., U14, U16, U18) in terms of the percentage of correct answers can be seen in Figure 4. According to the results, only the SPANT results showed significant differences among groups, with the U16 group outperforming the U14 and U18 groups by 4.5% and 6.4%, respectively (*p* = 0.01). Although there were no statistically significant differences in the remaining two tests, the results did show a visible trend that U18 players had the lowest percentages of correct answers in all three tests: (i) SIRT (U14 = 98.3 ± 2.9; U16 = 98.0 ± 4.4; U18 = 97.7 ± 3.2), (ii) CHORT (U14 = 92.5 ± 5.4; U16 = 93.9 ± 6.7; U18 = 91.4 ± 10.8), (iii) SPANT (U14 = 89.0 ± 11.9; U16 = 93.5 ± 5.5; U18 = 87.1 ± 9.2).

The comparison of psychomotor abilities according to the players’ position on the field (i.e., goalkeeper, center, left-back, right-back, left-wing, right-wing, pivot) is presented in Table 1. The results showed no significant differences in the performance of the three tests. However, some trends were observed in the results: (i) players who usually played on the right-wing were always the ones who had the highest number of correct answers in all three tests carried out (SIRT: 98.9% (*p* = 0.51, f = 0.1, small effect size); CHORT: 96.4% (*p* = 0.15, f = 0.23, small effect size); SPANT: 93.3% (*p* = 0.67, f = 0.17, small effect size)); (ii) center players performed better in two of the three tests in terms of movement time (CHORT: 198.8 ms (*p* = 0.44, f = 0.04, no effect size); SPANT: 233.9 ms (*p* = 0.83, f = 0.22, small effect size)); (iii) players who played pivot more often showed good reaction time, being in the top three of the tests carried out (SIRT: 314.7 ms (*p* = 0.25, f = 0.16, small effect size); CHORT: 622.2 ms (*p* = 0.52, f = 0.11, small effect size); SPANT: 600.4 ms (*p* = 0.65, f = 0.17, small effect size)); (iv) goalkeepers’ performance did not stand out in any of the conducted tests and abilities assessed (i.e., reaction time, movement time, percentage of correct answers).

**Table 1:** Descriptive statistics of psychomotor abilities and comparisons among groups according to players’ field positions.

Variables	GK (n = 11)	C (n = 13)	LB (n = 13)	RB (n = 8)	LW (n = 10)	RW (n = 9)	PV (n = 11)	Comparison between groups	
	Mean ± SD							*p*	f
SIRT RT (ms)	327.6 ± 25.1	336.9 ± 38.2	313.1 ± 32.1	333.6 ± 41.2	346.2 ± 36.3	330.9 ± 34.9	314.7 ± 24.1	0.25	0.16
SIRT MT (ms)	189.9 ± 40.5	190.5 ± 34.7	192.5 ± 41.5	206.3 ± 51.2	184.5 ± 18.3	199.3 ± 25.1	182.0 ± 48.1	0.73	0.19
SIRT CAs (%)	97.3 ± 6.1	98.9 ± 2.2	96.9 ± 3.3	98.1 ± 2.6	97.0 ± 4.2	98.9 ± 3.3	98.6 ± 2.3	0.51	0.1
CHORT RT (ms)	652.6 ± 42.6	640.7 ± 82.5	651.0 ± 67.8	656.4 ± 93.1	670.8 ± 49.0	661.0 ± 50.2	622.2 ± 74.2	0.52	0.11
CHORT MT (ms)	222.9 ± 87.5	198.8 ± 32.0	213.8 ± 41.4	239.3 ± 41.1	201.3 ± 29.7	208.4 ± 29.9	217.4 ± 50.3	0.44	0.04
CHORT CAs (%)	91.8 ± 7.0	89.3 ± 14.9	96.0 ± 3.7	87.1 ± 6.7	92.2 ± 6.1	96.4 ± 3.7	90.3 ± 6.4	0.15	0.23
SPANT RT (ms)	616.6 ± 80.1	595.1 ± 71.3	646.8 ± 108	606.1 ± 97.2	617.8 ± 94.8	575.3 ± 64.3	600.4 ± 130	0.65	0.17
SPANT MT (ms)	249.6 ± 47.0	233.9 ± 53.1	263.9 ± 71.1	267.4 ± 57.4	247.4 ± 65.2	250.7 ± 54.4	265.0 ± 59.3	0.83	0.22
SPANT CAs (%)	90.9 ± 6.6	91.2 ± 6.5	90.0 ± 11.0	91.3 ± 8.4	86.0 ± 10.2	93.3 ± 7.5	87.3 ± 12.9	0.67	0.17

SD (standard deviation), p (probability of testing), f (effect size), GK (goalkeeper), C (center), LB (left-back), RB (right-back), LW (left-wing), RW (right-wing), PV (pivot), SIRT (simple reaction times test), CHORT (choice reaction time test), SPANT (spatial anticipation test), RT (reaction time), MT (movement time), CAs (correct answers)

## Discussion

The primary purpose of this study was to characterize and analyze the psychomotor abilities of youth male handball players competing in different age groups (U14, U16, U18) and considering their field positions (goalkeeper, center, left-back, right-back, left-wing, right-wing, pivot). Although it was expected that older players (i.e., U18) would perform better regarding reaction time, movement time, and the percentage of correct answers, the results did not confirm it. The only statistically significant difference was in the percentage of correct answers in the test that assessed eye-hand coordination using spatial information (i.e., SPANT), showing that U16 players had a higher rate of correct answers than the others. Also, when comparing particular positions on the field, there were no statistically significant differences in reaction time, movement time, and the percentage of correct answers.

According to our results, U16 players showed a higher percentage of correct answers in the SPANT, which evaluated eye-hand coordination using spatial information. Still, this specific group performed better than U14 and U18 groups regarding reaction time in the three tests. Reaction time is interpreted as the interval between exposure to a stimulus and a muscle reaction (Đolo et al., 2022). Thus, these results were not expected as scientific research has shown that practicing sports and belonging to more advanced age groups has been associated with better reaction time values (i.e., better performance) than in younger players (Akarsu et al., 2009; Millard et al., 2021; Śliż et al., 2022). Therefore, it would be expected that the U18 players would be quicker to react to a stimulus and have a more accurate percentage of correct answers than U16 players. Again, previous studies have verified that older handball players typically performed better regarding eye-hand coordination evaluated using spatial information (Akarsu et al., 2009; Millard et al., 2021; Śliż et al., 2023). On the other hand, U18 players showed better performance in two out of the three tests related to movement time. Movement time is interpreted as the duration of a move after the response to the initial reaction to the stimulus (Đolo et al., 2022). Regarding movement time, the results align with the literature above (Akarsu et al., 2009; Millard et al., 2021; Śliż et al., 2023). However, it is to be expected that the U18 training and competition regime will be programmed with greater intensity, decision-making exercises with shorter times and greater complexity. It was, therefore, expected that reaction time and the percentage of correct answers would be significantly better at the U18 level, something that did not occur.

No statistically significant differences were found in our study regarding psychomotor abilities according to players’ field positions. However, some trends could be noticed. Players who primarily played on the right-wing had more correct answers over the three tests. Interestingly, all those players had their left upper limbs as preferred. According to the literature, psychomotor abilities, such as those studied in this research, are influenced by many factors, including the dominant hand (Dane and Erzurumluoglu, 2003). Also, an investigation conducted with 40 professional handball players in the Polish Super League concluded that wing players recorded better movement times in the SIRT (Przednowek et al., 2019) which is a psychomotor test assessing reaction speed and stability (Tarnowski, 2014).

Still, goalkeepers in this sample did not stand out in any of the tests or psychomotor skills assessed. A study that aimed to investigate handball players’ cognitive abilities verified that goalkeepers committed fewer errors than pivot and back players when performing the cognition test within the Vienna Test System (Kiss and Balogh, 2019). According to another investigation with 35 national team handball players, goalkeepers were found to have faster reaction times than the other field players (Blecharz et al., 2022). Goalkeepers are expected to be brave, agile, and flexible and to have a high pain threshold (Silva, 2006). Furthermore, goalkeepers must react quickly to shifting circumstances, relying on their reflexes and decision-making abilities (Blecharz et al., 2022). It was expected that goalkeepers in this sample would stand out regarding reaction and movement time compared to their colleagues in other positions. Thus, these results reinforce that training for this position should be adjusted in the future, with specific exercises for cognitive development and stimulation, aiming to complement physical conditioning with psychomotor training.

Reflecting on all the presented results, the handball rule changes, which aim to increase the general pace of the game, unpredictability, goal-scoring opportunities, shorter decision-making times, and faster game readings, are critical factors for individual and collective sporting success (Smolarczyk, 2023). This means that minor differences, many of them statistically undetectable, can determine whether a player takes advantage of a clear opportunity in handball and in other team sports.

This study has some limitations that should be considered when analyzing the results. Although the number of players per competitive age group and per position on the court was balanced, a larger sample would bring more consistency and breadth to the results presented. Therefore, some caution is recommended when interpreting and extending the results to other populations of handball players. Furthermore, the need for more information on body composition, physical fitness, and maturational status limits our research. This information would provide a more in-depth analysis of group differences. However, this study has novelty value and presents a primary approach to comparing handball players from different age groups. The successive changes to handball regulations over the last decade have aimed to make the game faster, more spectacular, and with more apparent scoring opportunities. As a result, reaction, movement, and decision-making times need to become shorter and shorter if players are to succeed in their actions, regardless of their age group or position on the court.

## Conclusions

The results obtained in this study show no statistically significant differences in reaction time, movement time, and the percentage of correct answers among players of U14, U16, and U18 age categories. Also, there were no notable differences considering their positions on the field. Only U16 players showed significantly better results in the SPANT regarding the rate of correct answers. Practically reflecting, slight differences in those psychomotor abilities can significantly affect individual and collective success or failure in handball and team sports. Handball is one of the fastest team sports, with players having less and less time to make decisions, thus coaches and their staff should prioritize and implement developing psychomotor abilities in their training programs, regardless of the age group and a field position. Future research should consider youth players’ maturation stage, body composition, and physical fitness to characterize their profile better and analyze their psychomotor abilities, taking into account other factors that impact their performance and development.
